# STIM1-dependent store-operated calcium entry mediates sex differences in macrophage chemotaxis and monocyte recruitment

**DOI:** 10.1016/j.jbc.2024.107422

**Published:** 2024-05-28

**Authors:** Adriana M. Fresquez, James O. Hogan, Patricia Rivera, Kristen M. Patterson, Kanakadurga Singer, Joseph M. Reynolds, Carl White

**Affiliations:** 1 Physiology & Biophysics, Center for Cancer Cell Biology, Immunology, and Infection, Rosalind Franklin University of Medicine & Science, North Chicago, Illinois, USA; 2 Microbiology and Immunology, Center for Cancer Cell Biology, Immunology, and Infection, Rosalind Franklin University of Medicine & Science, North Chicago, Illinois, USA; 3 Department of Pediatrics, Michigan Medicine, University of Michigan, Ann Arbor, Michigan, USA

**Keywords:** stromal interaction molecule 1, Orai, C5a, migration, calcium, CRAC channel

## Abstract

Infiltration of monocyte-derived cells to sites of infection and injury is greater in males than in females, due in part, to increased chemotaxis, the process of directed cell movement toward a chemical signal. The mechanisms governing sexual dimorphism in chemotaxis are not known. We hypothesized a role for the store-operated calcium entry (SOCE) pathway in regulating chemotaxis by modulating leading and trailing edge membrane dynamics. We measured the chemotactic response of bone marrow-derived macrophages migrating toward complement component 5a (C5a). Chemotactic ability was dependent on sex and inflammatory phenotype (M0, M1, and M2), and correlated with SOCE. Notably, females exhibited a significantly lower magnitude of SOCE than males. When we knocked out the SOCE gene, stromal interaction molecule 1 (STIM1), it eliminated SOCE and equalized chemotaxis across both sexes. Analysis of membrane dynamics at the leading and trailing edges showed that STIM1 influences chemotaxis by facilitating retraction of the trailing edge. Using BTP2 to pharmacologically inhibit SOCE mirrored the effects of STIM1 knockout, demonstrating a central role of STIM/Orai-mediated calcium signaling. Importantly, by monitoring the recruitment of adoptively transferred monocytes in an *in vivo* model of peritonitis, we show that increased infiltration of male monocytes during infection is dependent on STIM1. These data support a model in which STIM1-dependent SOCE is necessary and sufficient for mediating the sex difference in monocyte recruitment and macrophage chemotactic ability by regulating trailing edge dynamics.

Sexual dimorphism in the innate immune system is well-established and determined by a complex interaction between hormones, the environment, and genetics (1, 2). Macrophages in particular have been identified as being intrinsically different between males and females (1). One key difference is the increased infiltration of macrophages to sites of infection and injury in males compared to females (3, 4, 5, 6). Infiltrating macrophages are derived from circulating monocytes and recruited during inflammation by chemotaxis, the process of directed migration along a concentration gradient of chemoattractant protein (7). Macrophages are also highly plastic, and their inflammatory phenotype is complex and likely exists along a continuum of proinflammatory (M1 or classically activated) to anti-inflammatory (M2 or alternatively activated) subtypes. Importantly, once at the site of inflammation, macrophages adopt a position along the M1-M2 spectrum that is determined by sex as well as the local environment (5, 8, 9). Interestingly, M1-polarized macrophages demonstrate less robust chemotactic ability (10, 11, 12). While considerable effort has been focused on defining the regulation of macrophage phenotype and the mechanisms of chemotaxis, the influence of biological sex on how these pathways interact has not been explored.

Chemotactic motility progresses by a series of steps beginning with the extension of lamellipodia at the cell’s leading edge, followed by substrate attachment at focal adhesion complexes, and ending in forward movement initiated by the coordinated contraction of actin filaments and detachment of the trailing edge (13). These steps are tightly regulated by distinct spatial and temporal cytoplasmic calcium (Ca^2+^) signals evoked by chemoattractant-receptor interactions (14, 15, 16, 17, 18). Several types of Ca^2+^ channels have been identified to play a role in this process although it isn’t known if their expression or function is affected by sex and inflammatory phenotype.

The store-operated calcium entry (SOCE) pathway is ubiquitous and impinges on diverse cellular processes (19, 20), and has been implicated as a regulator of motility in many cell types including lymphocytes (14, 21, 22, 23, 24, 25). SOCE is activated following Ca^2+^ release from the endoplasmic reticulum (ER) Ca^2+^ stores. Decreased store content is sensed by the ER membrane-resident STIM1 and 2 proteins which then interact and gate open the plasma membrane Ca^2+^ release-activated Ca^2+^ (CRAC) channels Orai1, 2 and 3. Depending on the degree of activation, the resultant Ca^2+^ influx can serve to locally refill the store or support larger global Ca^2+^ signals (19). Knockdown and overexpression studies in cell lines have clearly demonstrated a role for STIM and Orai proteins in determining migration efficiency, most notably in cancer cells (26). Mechanistically, STIM and Orai-mediated SOCE have been shown to influence chemotaxis by regulating lamellipodia formation as well as focal adhesion turnover at both the leading and trailing edges (21, 27, 28); however, it remains to be determined if these mechanisms are operative in macrophages.

The overall objective of the current study was to gain mechanistic insight into how SOCE-mediated Ca^2+^ signaling might interact with biological sex to influence monocyte recruitment and macrophage chemotaxis. We hypothesized that Ca^2+^ signaling by SOCE is a key regulator of chemotaxis whose modulation of leading and trailing edge membrane dynamics determines sex differences and dictates how cells respond to inflammatory stimuli. This hypothesis was tested using bone marrow-derived macrophages (BMDMs) from both male and female, wild-type and STIM1-knockout mice, by examining the influence of sex and inflammatory phenotype on SOCE, STIM, and Orai gene expression, and *in vitro* and *in vivo* chemotaxis.

## Results

### Sex and inflammatory phenotype interact to determine macrophage chemotaxis and calcium signaling

**Figure 1:**
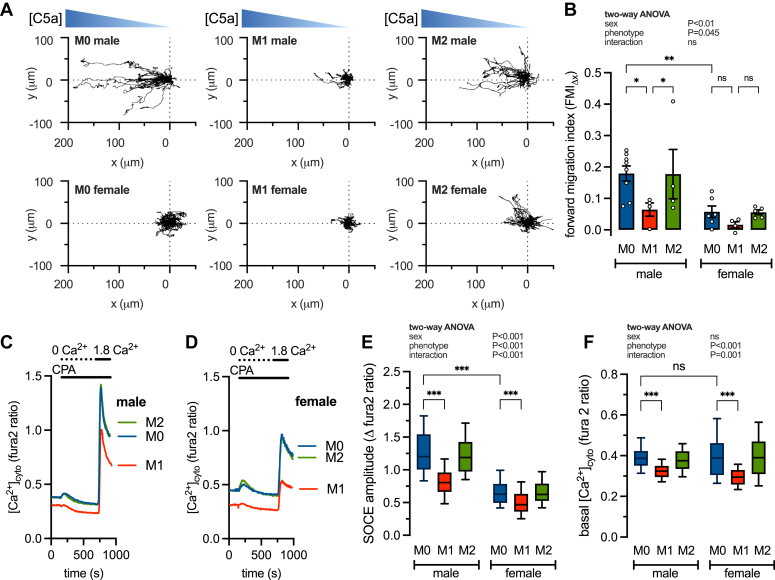
**Sex and inflammatory phenotype determine differences in chemotaxis, basal cytoplasmic [Ca**^**2+**^**] ([Ca**^**2+**^**]**_**cyto**_**), and store-operated calcium entry (SOCE).***A*, representative migration tracks of male and female M0, M1, and M2 bone marrow-derived macrophages (BMDMs) in a C5a gradient. *B*, summary (mean ± SEM) of the mean forward migrating index (FMI_Δx_). Each data point represents measurements from 85 to 448 cells derived from 5 male and 7 female mice and pooled from 4 to 8 independentnt experiments. ∗∗*p* < 0.01, ∗*p* < 0.05 (2-way ANOVA). *C* and *D*, representative [Ca^2+^]_cyto_ recordings in male and female M0, M1, and M2-treated bone marrow-derived macrophages (BMDMs). *E* and *F*, summary data showing SOCE amplitude and basal [Ca^2+^]_cyto_. Each dataset represents measurements from 90 to 207 cells derived from 4 male and 3 female mice and pooled from 4 to 7 independent experiments. ∗∗∗*p* < 0.001 (two-way ANOVA).

We first assessed the interaction between sex and inflammatory phenotype on bone marrow-derived macrophage (BMDM) chemotaxis. To generate proinflammatory (M1) and anti-inflammatory (M2) phenotypes we treated cells for 24 h with LPS/IFNγ (100 ng/ml and 50 ng/ml) or IL-4 (10 ng/ml), respectively (29, 30). Untreated, non-activated (M0) BMDMs served as controls. We then loaded cells into a μ-Slide chemotaxis assay chamber (31) and used phase-contrast video microscopy to track movement along a concentration gradient of the chemoattractant, complement component 5a (C5a; 20 nM) (Supplementary Videos 1 and 2) (32, 33). Figure 1*A* shows the individual migration paths for male and female M0, M1, and M2 cells. To better quantify the efficiency and directionality of cell movement we calculated the Forward Migration Index (FMI_Δx_) for individual cells. FMI is the ratio between the cell’s progress in the correct direction (a straight line toward the stimulus) and the actual path that the cell travels. Here, a value closer to 1 indicates the cell is moving directly toward the chemoattractant, whereas a value closer to 0 indicates that migration is unrelated to the chemoattractant. We calculated the FMI_Δx_ for each cell and summarized the mean for each experimental replicate (Fig. 1*B*). Chemotaxis in male cells, as measured by FMI_Δx_, is highest in M0 and M2 cells and inhibited by M1 stimulation. In comparison, female cells are only weakly chemotactic and chemotaxis is completely eliminated by M1 stimulation (Fig. 1*B*). These data show that chemotaxis is highly dependent on both sex and inflammatory phenotype.

Store-operated calcium entry (SOCE) was shown to regulate motility and chemotaxis in neutrophils and dendritic cells (23, 34). We postulated that changes in SOCE might underpin the effects of sex and inflammatory phenotype on BMDM chemotaxis. To test this, we measured the magnitude of maximally activated SOCE in M0, M1, and M2 macrophages. To do this we first depleted the endoplasmic reticulum (ER) stores in the absence of extracellular Ca^2+^ by inhibiting the sarco-endoplasmic reticulum Ca^2+^-ATPase (SERCA) pump with the blocker cyclopiazonic acid (CPA; 10 ***μ***M). When Ca^2+^ is added back to the media it enters the cell through the activated Ca^2+^ release-activated Ca^2+^ (CRAC) channels—the amplitude of the resulting increase in [Ca^2+^]_cyto_ therefore reflects the activation status of SOCE. The effect of phenotype was qualitatively similar in both males and females, in that, when compared to M0, M1 stimulation reduced SOCE and M2 had no effect on SOCE (Fig. 1, *C* and *D*). Surprisingly, the amplitude of SOCE in unstimulated M0 cells from females was significantly lower than that of males (Fig. 1*E*). In addition, basal [Ca^2+^]_cyto_ was the same in male and female M0 and M2 cells and decreased by M1 stimulation (Fig. 1*F*).

### Sex and inflammatory phenotype regulate the expression levels of STIM and Orai isoforms

**Figure 2:**
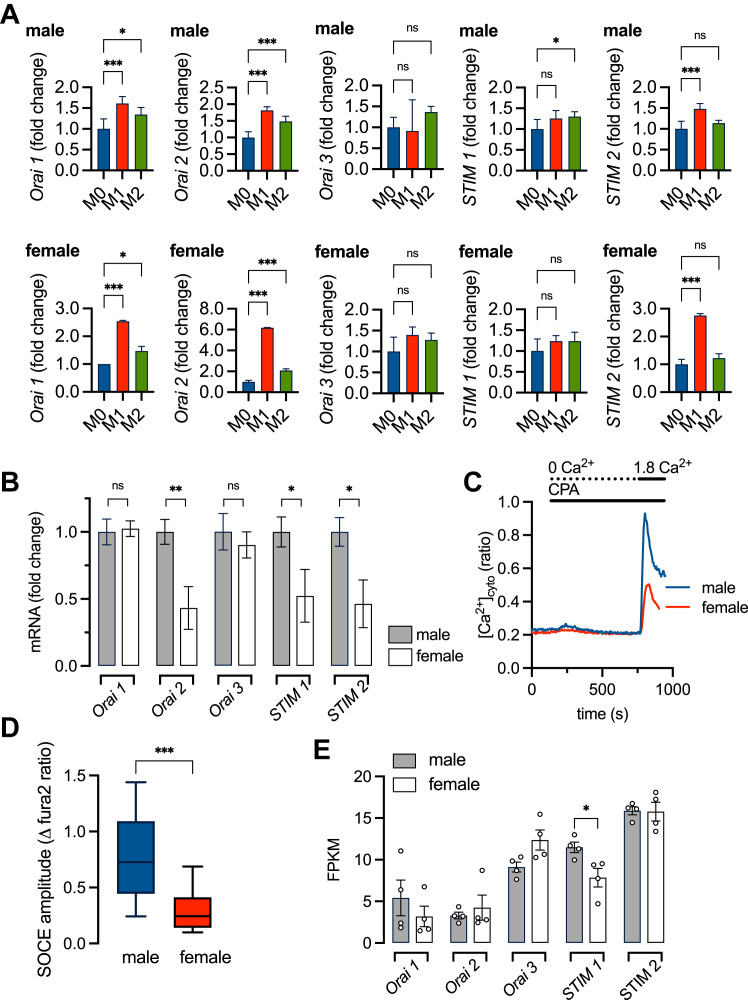
**Sex and inflammatory phenotype regulate *STIM* and *Orai* gene expression.***A*, expression of *STIM1, 2* and *Orai1, 2* and *3* genes in male and female bone marrow-derived macrophages (BMDMs) determined by quantitative real-time PCR. Data (mean ± SD) are pooled from 5 male and 5 female mice, ∗∗∗*p* < 0.001, ∗*p* < 0.05 (one-way ANOVA). *B*, expression of *STIM1, 2* and *Orai1, 2* and *3* genes in BMDMs plotted as female relative to male (∗∗*p* < 0.01, ∗*p* < 0.05; Student’s *t* test). *C*, original traces showing SOCE-dependent [Ca^2+^]_cyto_ influx in freshly isolated male and female adipose tissue macrophages (ATMs). *D*, summary data showing SOCE amplitude in male (n = 204 cells, 9 experiments, 3 animals) and female (n = 67 cells, 7 experiments, 3 animals) ATMs (∗∗∗*p* < 0.001; Student’s *t* test). *E*, summary (mean ± SEM) of RNA-seq data plotted as FPKM (fragments per kilobase of exon model per million reads mapped) showing gene expression of *STIM1, 2* and *Orai1, 2* and *3* in male (n = 4 animals) and female (n = 4 animals) ATMs. NCBI GEO accession GSE181841 (∗*p* < 0.05; Student’s *t* test).

To further explore the mechanisms of sex and phenotypic-dependent changes in SOCE amplitude we measured the expression levels of *STIM* and *Orai* genes. Using qRT-PCR, we quantified the mRNA levels of *Orai1*, *2* and *3*, and *STIM1* and *2* in M0, M1, and M2 bone marrow-derived macrophages (BMDMs) isolated from males and females. The effects of phenotype on *STIM* and *Orai* isoforms were highly consistent between the sexes. In both male and females, M1 and M2 polarization induced upregulation of *Orai1* and *2*, while *STIM2* was upregulated in M1 cells only (Fig. 2*A*). M2 stimulation did statistically increase *STIM1* expression in males only although the trend was the same in females. Overall, our data are consistent with the hypothesis that inflammatory phenotype remodels SOCE similarly in males and females by altering the expression of specific *STIM* and *Orai* isoforms. We next asked if the differential expression of *STIM* and *Orai* isoforms could account for the reduced SOCE seen in females. We compared *STIM* and *Orai* expression in females relative to male M0 cells and found that *Orai2*, *STIM1*, and *STIM2* were expressed at significantly lower levels in females (Fig. 2*B*). To confirm that sex differences in SOCE are not experimental artifacts of differentiated BMDMs we measured SOCE in freshly isolated male and female primary adipose tissue macrophages (ATMs). Similar to BMDMs, the magnitude of SOCE was smaller in females compared to males (Fig. 2, *C* and *D*). Transcriptomic analysis of publicly available datasets (NCBI GEO accession GSE181841) (5) revealed decreased levels of *STIM1* in ATMs from females compared to males (Fig. 2*E*). Taken together, these data show that the decreased SOCE phenotype is associated with remodeling of *STIM/Orai* gene expression.

### Sex differences in C5a-dependent [Ca^2+^]_cyto_ signaling and chemotaxis are STIM1-dependent

**Figure 3:**
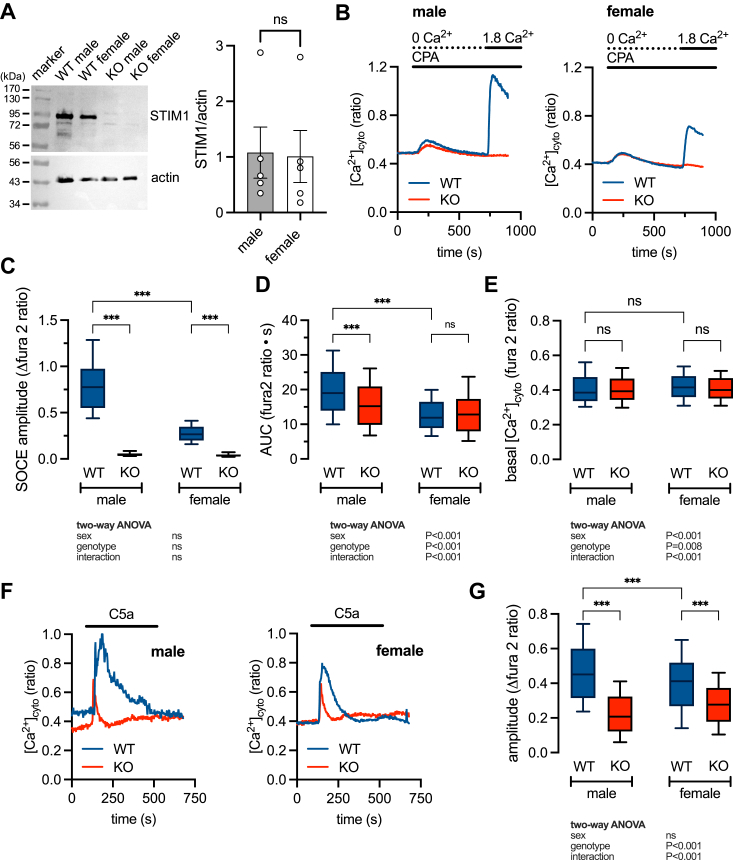
**Knocking out STIM1 abolishes store-operated Ca**^**2+**^**entry (SOCE) and attenuates C5a-evoked [Ca**^**2+**^**]**_**cyto**_**transients.***A*, Western blot (*left-hand panel*) showing expression of STIM1 in WT and STIM1-KO (KO) bone marrow-derived macrophages (BMDMs) from male and female. Summary (mean ± SEM) of densitometry analysis (*right-hand panel*) from 5 male and 5 female WT animals (Student’s *t* test). *B*, representative [Ca^2+^]_cyto_ recordings in male and female, WT and STIM1-KO (KO) BMDMs, showing store depletion in zero external Ca^2+^ and the resultant Ca^2+^ influx through the maximally activated SOCE pathway. *C–E*, summary data showing SOCE amplitude (*C*), ER content estimated by area under the curve of the CPA-evoked transient (*D*), and basal [Ca^2+^]_cyto_ (*E*). Each dataset represents measurements from 119 to 155 cells derived from 3 male WT and 3 female KO mice and pooled from 5 to 6 independent experiments. *F* and *G*, representative [Ca^2+^]_cyto_ recordings in male and female, WT and KO BMDMs, showing transient increases in [Ca^2+^]_cyto_ in response to addition of C5a (20 nM). Summary data show the [Ca^2+^]_cyto_ transient amplitude evoked by C5a. Each dataset represents measurements from measurements in 105 to 168 cells derived from 3 male WT and 3 female KO mice and pooled from 5 to 7 independent experiments. ∗∗∗*p* < 0.001 (two-way ANOVA).

Our Ca^2+^ imaging data show that macrophages from females have a lower SOCE compared to males and that SOCE can be further inhibited in both males and females by M1 stimulation. Importantly, decreased SOCE repeatedly correlates with decreased chemotaxis regardless of sex and phenotype. To establish causation, we knocked out the *STIM1* gene in cells of the hematopoietic lineage by crossing STIM1^fl/fl^ mice with Vav-iCre to generate Vav-cre^−^STIM1^fl/fl^ control (WT) and Vav-cre^+^STIM1^fl/fl^ (STIM1-KO). The deletion was confirmed by the complete loss of STIM1 protein (Fig. 3*A*) and SOCE (Fig. 3, *B* and *C*) in unstimulated (M0) BMDMs from both males and females. Interestingly, the abundance of STIM1 protein is the same in males and females (Fig. 3*A*), indicating that sex differences in the magnitude of SOCE are not due to differential STIM1 protein expression.

The transient increase in [Ca^2+^]_cyto_ seen upon CPA (10 ***μ***M) application reflects the release of stored ER Ca^2+^, and so measuring the area under the curve yields an indirect measure of the filling state of the ER. STIM1 knockout decreased the ER content in males but not females indicating a dependency on SOCE to maintain the filling state in male BMDMs (Fig. 3*D*). Basal [Ca^2+^]_cyto_ was unaffected by loss of *STIM1* (Fig. 3*E*). Binding of C5a to its G-protein coupled receptor increases [Ca^2+^]_cyto_ by triggering IP_3_R-dependent ER Ca^2+^ release and SOCE activation (35, 36, 37). However, it is not known if there is a sex difference in the response to C5a. Exposing WT BMDMs to C5a evoked a transient increase in [Ca^2+^]_cyto_ which was larger in male cells compared to female (Fig. 3, *F* and *G*). Furthermore, the [Ca^2+^]_cyto_ response to C5a was similarly attenuated by STIM1 knockout in both males and females (Fig. 3, *F* and *G*), demonstrating that the sex difference in C5a response can be attributed to STIM1-dependent SOCE.

**Figure 4:**
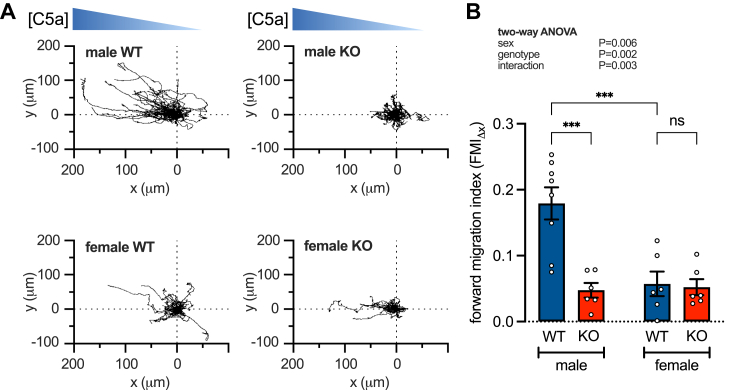
**Knocking out STIM1 inhibits chemotaxis in males.***A*, representative migration tracks of male and female, WT and STIM1-KO (KO) bone marrow-derived macrophages (BMDMs) in a C5a gradient. *B*, summary (mean ± SEM) of the mean forward migrating index (FMI_Δx_). Each data point represents measurements from 231 to 448 cells derived from male WT (n = 5), male KO (n = 3), female WT (n = 5), and female KO (n = 3) mice and pooled from 4 to 8 independent experiments. ∗∗∗*p* < 0.001 (2-way ANOVA).

We then measured C5a-stimulated chemotaxis in M0 WT and STIM1-KO BMDMs from males and females using the μ-Slide assay chamber (Fig. 4*A*). The Forward Migration Index (FMI_Δx_) is summarized in Figure 4*B* and shows that knockout of STIM1 normalized chemotaxis in male and female BMDMs by reducing the chemotactic ability of male cells. Thus, sex differences in chemotaxis are STIM1-dependent.

### Sex differences at the trailing but not leading edges are STIM1-dependent

**Figure 5:**
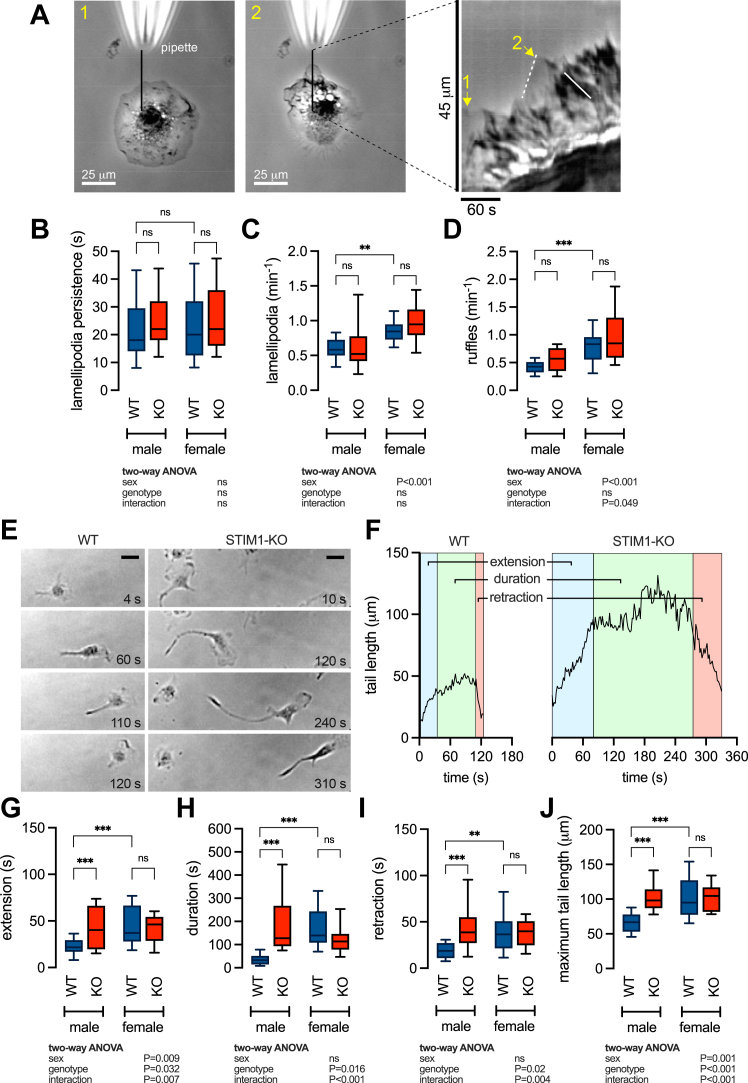
**Knocking out STIM1 has no effect on sex differences at the leading edge but eliminates sex differences in trailing edge dynamics.***A*, images of bone marrow-derived macrophages (BMDMs) captured before (t = 0) and during (t = 140s) application of C5a from the pipette. The solid line shows the position of the 1-pixel-wide image extracted from each frame and aligned to form the space-time plot. Lamellipodia protrusions are represented by linear ascending contours (*white dashed line*), with their persistence measured by the duration these contours last before they start to retract. Ruffle frequency is determined by counting the number of dark descending lines (*white solid line*), which represent membrane ruffles, per unit of time. *B–D*, summary data showing lamellipodia persistence (*B*), lamellipodia (*C*) and ruffle (*D*) frequency. Each dataset contains measurements from 72 to 139 cells derived from male WT (n = 4), female WT (n = 4), male KO (n = 5), and female KO (n = 3) mice and pooled from 9 to 11 independent experiments. ∗∗∗*p* < 0.001 (two-way ANOVA). *E*, representative images of male WT and KO BMDMs at different time points as they migrate in a C5a gradient. The direction of motion is *left to right* and each set of images depicts one extension, duration, and retraction cycle of the trailing edge tail. Scale bar = 25 μm. *F*, the time course of tail extension (*blue shading*), duration (*red shading*), and retraction (*green shading*) in representative WT and STIM1-KO cells. *G–J*, summary data of extension, duration, and retraction phases as well as the maximum tail length during one cycle. Data are measurements from 20 to 22 cells derived from male WT (n = 5), female WT (n = 7), male KO (n = 5), and female KO (n = 4) mice and pooled from 6 to 10 independent experiments. ∗∗∗*p* < 0.001, ∗∗*p* < 0.01 (two-way ANOVA).

Directed motility requires the coordination of leading and trailing edge membrane dynamics (38); therefore, we postulated that STIM1-mediated SOCE regulates directionality by influencing membrane dynamics. To assess dynamics at the leading edge, we exposed cells to a highly localized C5a gradient delivered from a micropipette and visualized the formation of lamellipodia using phase contrast video microscopy. We were also able to discern membrane ruffles, which form when lamellipodia fail to adhere and detach from the substrate and could be observed as waves of dark contrast moving away from the leading edge (Fig. 5*A*, and Supplementary Video 3). We quantified lamellipodia persistence as well as lamellipodia and ruffle frequency from kymograph plots of the time-lapse recordings (Fig. 5, *B–D*). While STIM1 knockout did not affect any of the measured variables, there was a significant effect of sex on the frequency of lamellipodia and ruffles (Fig. 5, *C* and *D*). Increased frequency of lamellipodia formation and ruffling can be attributed to decreased substrate adherence and loss of efficient migration (39). Taken together, these data suggest a role for leading-edge dynamics in determining sex differences but cannot account for STIM1-dependent effects on chemotaxis.

To examine the role of STIM1 in regulating trailing edge dynamics we performed a secondary image analysis of WT and STIM1-KO cells migrating in the μ-Slide assay chamber (Fig. 5, *E* and *F*). A key step in forward motion is the disassembly of adhesions and retraction of the trailing edge. A single cycle capturing the formation, maintenance, and retraction of the trailing edge for a representative WT and STIM1-KO cell is shown (Fig. 5*E*). To quantify this process, we monitored the change in tail length, defined as the distance along the trailing edge from the center of the cell body to the tip of the tail (Fig. 5*F*). The extension and retraction phases were quantified by measuring the rise and fall times required for the tail to transition from 5% to 95% of its final maximum and minimum lengths, respectively. These data are summarized together with measurements of tail duration and maximum tail length (Fig. 5, *G–F*). Tail extension, duration, and retraction times as well as maximum tail length were all longer in females compared with males. All of these metrics of trailing edge function were increased by STIM1 knockout in males but were unaffected by STIM1-KO in females (Fig. 5, *G–F*). These data demonstrate the dependence of trailing edge dynamics on sex and STIM1 and support a role for SOCE in mediating sex differences in chemotaxis by regulating Ca^2+^ signaling at the trailing edge.

### Pharmacological inhibition of SOCE attenuates macrophage chemotaxis and trailing edge retraction

**Figure 6:**
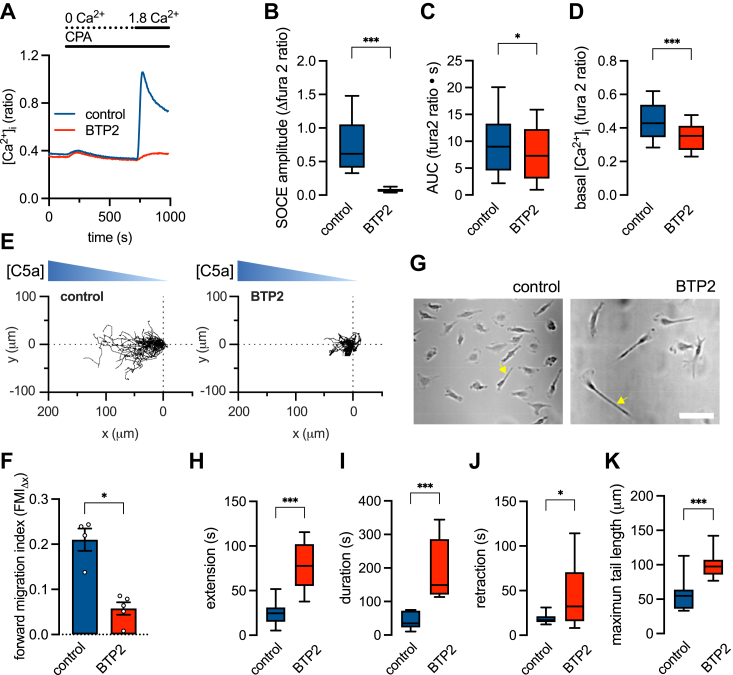
**The store-operated Ca**^**2+**^**entry (SOCE) blocker, BTP2, inhibits chemotaxis and modifies trailing edge dynamics in male WT cells.***A*, representative [Ca^2+^]_cyto_ recordings in male WT bone marrow-derived macrophages (BMDMs). Traces show store depletion in zero external Ca^2+^ and the resultant Ca^2+^ influx through the maximally activated SOCE pathway in cells pretreated with either BTP2 or vehicle control. *B–D*, summary data showing SOCE amplitude (*B*), ER content estimated by area under the curve of the CPA-evoked transient (*C*), and basal [Ca^2+^]_cyto_ (*D*). Datasets represent measurements from 123 to 137 cells derived from 3 male WT mice and pooled from 5 to 6 independent experiments. ∗∗∗*p* < 0.001, ∗*p* < 0.05 (Student’s *t* test). *E*, representative migration tracks of WT male cells pretreated with BTP2 (10 μM) or vehicle control moving in a C5a gradient. *F*, summary (mean ± SEM) of the mean forward migrating index (FMI_Δx_) from 100 to 251 cells derived from 3 male WT mice and pooled from 4 to 6 independent experiments, ∗*p* < 0.05 (Student’s *t* test). *G*, representative images of male WT BMDMs migrating in a C5a gradient. Arrows indicate trailing edge tails and the scale bar = 50 μm. *H–K*, summary data of extension, duration, and retraction phases, as well as the maximum tail length during one cycle. Data are measurements from 11 to 12 cells derived from male WT (n = 5), mice and pooled from 4 to 5 independent experiments. ∗∗∗*p* < 0.001, ∗*p* < 0.05 (Student’s *t* test).

To confirm that STIM1-KO phenotypes are truly Ca^2+^ signaling dependent, we assessed the effects of BTP2, an Orai channel inhibitor and blocker of SOCE, on chemotaxis in male BMDMs. Preincubation (1 h) with BTP2 (1 μM) blocked SOCE (Fig. 6, *A* and *B*) as well as decreased ER store content (Fig. 6*C*). BTP2 also decreased [Ca^2+^]_cyto_ (Fig. 6*D*), consistent with its known effects on other Ca^2+^ regulatory pathways (40). As with STIM1 knockout, BTP2 decreased macrophage chemotaxis (Fig. 6, *E* and *F*) by modulating trailing edge dynamics (Fig. 6, *G–K*). These data further support the hypotheses that STIM1 regulates chemotaxis through modulation of SOCE-mediated Ca^2+^ signaling.

### STIM1 determines sex differences in monocyte recruitment *in vivo*

**Figure 7:**
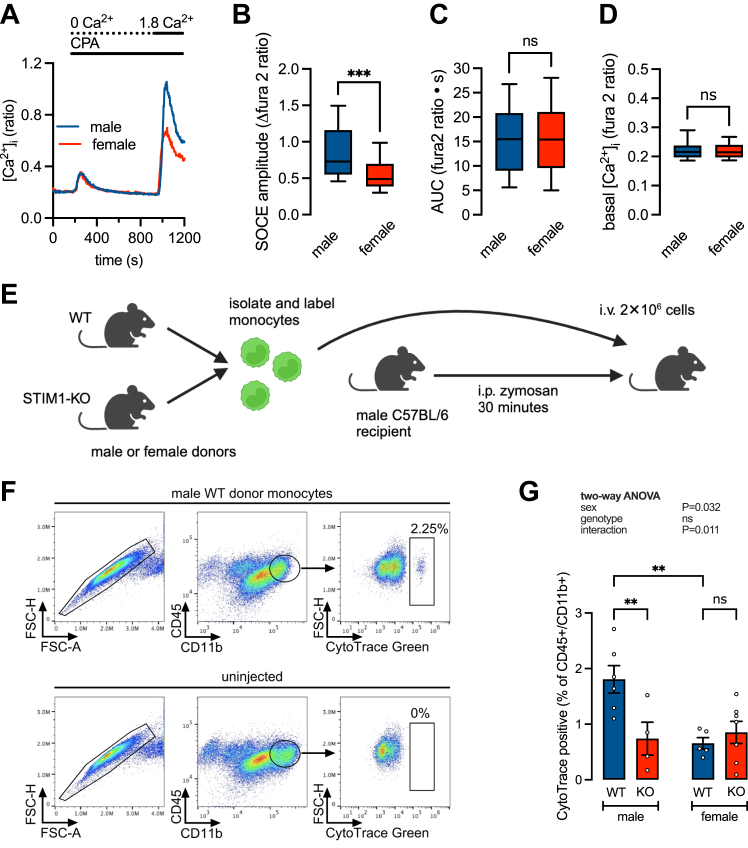
**STIM1 knockout in adoptively transferred monocytes decreases the recruitment of male but not female cells during acute inflammation *in vivo*.***A*, representative [Ca^2+^]_cyto_ recordings in WT male and female bone marrow-derived monocytes, showing store depletion in zero external Ca^2+^ and the resultant Ca^2+^ influx through the maximally activated SOCE pathway. *B–D*, summary data showing SOCE amplitude (*B*), ER content estimated by area under the curve of the CPA-evoked transient (*C*), and basal [Ca^2+^]_cyto_ (*D*). Each dataset represents measurements from 199 to 225 cells derived from 4 male and 3 female mice and pooled from 11 independent experiments. ∗∗∗*p* < 0.001 (two-way ANOVA). *E*, donor monocytes from the bone marrow of WT and STIM1-KO, male and female mice were labeled with CytoTrace green and i.v. injected into recipient (wild type male C57BL/6) mice 30 min after the induction of peritonitis by i.p administration of zymosan (1 mg/ml). Peritoneal cells were harvested after 18 h and analyzed by flow cytometry. Created with BioRender.com. *F*, flow cytometry plots from a representative experiment quantifying the percentage of adoptively transferred (male WT) CytoTrace positive cells in the monocyte/macrophage population characterized as CD45+CD11b+. *G*, summary data (mean ± SEM) showing the number of CytoTrace positive cells as a percentage of the peritoneal CD45+/CD11b+ population. Monocytes from single donors (n = 4–7 mice) were adoptively transferred into a single recipient, ∗∗∗*p* < 0.001 (two-way ANOVA).

Circulating bone marrow-derived monocytes are the primary source of macrophages recruited to the tissue in response to inflammatory stimulation. We isolated bone marrow-derived monocytes from male and female WT and measured SOCE. The magnitude of SOCE was lower in females compared to males (Fig. 7, *A* and *B*) while both basal [Ca^2+^]_cyto_ and ER store content were the same (Fig. 7, *C* and *D*). To determine the role of STIM1 in monocyte infiltration we measured the recruitment of adoptively transferred monocytes *in vivo* using the well-characterized zymosan model of peritonitis (41) First, we isolated and purified monocytes from the bone marrow of male or female WT or STIM1-KO mice and labeled them with the cell tracer CytoTrace Green CMFDA. Next, labeled donor monocytes were i.v.-injected into male C57BL/6J recipients previously i.p.-injected with the inflammatory stimulus zymosan (1 mg/ml). Finally, peritoneal cells were harvested at 18 h and labeled with CD45 and CD11b antibodies as markers of the total monocyte/macrophage population (Fig. 7*E*). The recruited CytoTrace-positive donor cells were quantified by flow cytometry as the percentage of the total macrophage population using the gating scheme depicted in Figure 7*F*. Consistent with previous studies, the recruitment of WT male monocytes was greater than female monocytes. Additionally, the knockout of STIM1 decreased the recruitment of male monocytes only (Fig. 7*G*). These observations are consistent with our *in vitro* chemotaxis data and support a necessary role for STIM1 in mediating sex differences in monocyte/macrophage recruitment *in vivo*.

## Discussion

The current study tested the hypothesis that Ca^2+^ signaling by SOCE is a key regulator of macrophage chemotaxis whose modulation of leading and trailing edge membrane dynamics determines sex differences and dictates how cells respond to inflammatory stimuli. The major findings are: (1) BMDMs from males are more chemotactic than those from females; (2) chemotaxis in males is inhibited by a switch to pro-inflammatory M1 phenotype; (3) SOCE is larger in male BMDMs and adipose tissue macrophages compared to females and is inhibited by M1 stimulation; (4) Knockout of STIM1 abolishes SOCE in male and female BMDMs and eliminates the sex difference in chemotaxis; (5) STIM1 mediates sex differences in trailing edge dynamics but has no effect on leading edge membrane dynamics; and (6) STIM1 mediates sex differences in monocyte recruitment during peritonitis. Collectively, our data support a model in which sex and phenotypic modulation of chemotaxis are tightly controlled by STIM1-dependent regulation of trailing edge dynamics.

Increased chemotactic ability in male compared with female cells has been reported for monocytes and macrophages (4, 5), neutrophils (3), and microglia (42), and reproduced by the current study (Fig. 1, *A* and *B*). It is well known that transition to the M1 phenotype robustly inhibits macrophage chemotaxis (10, 12, 43, 44, 45). We induced M1 activation using the standard protocol of treating cells for 24 h with LPS/IFNγ and showed decreased chemotaxis (Fig. 1*B*). A similar trend was observed in the female, although not significant, suggesting that phenotypic-induced changes in chemotaxis operate over a smaller range in the female. Physiologically, less chemotactic ability could enable female macrophages to persist longer at sites of infection and contribute to the more aggressive immune responses seen in females (2). Interestingly, acute exposure to LPS increases chemotaxis in neutrophils, dendritic cells, and monocytes (46, 47, 48); however, this disparity is likely due to these cells having different functional requirements as well as the shorter duration of LPS exposure.

We have identified novel sex differences in macrophage [Ca^2+^]_cyto_ signaling. We found that M1 stimulation decreased SOCE and basal [Ca^2+^]_cyto_ in both male and female BMDMs (Fig. 1, *C–E*). The effects of phenotype on SOCE and basal [Ca^2+^]_cyto_ are likely unrelated for several reasons. First, SOCE is also lower in females compared to males, regardless of phenotype (Fig. 1*E*), and second, complete ablation of SOCE by STIM1 knockout does not affect basal [Ca^2+^]_cyto_ (Fig. 3*E*). The mechanism by which M1 stimulation lowers basal [Ca^2+^]_cyto_ remains unknown and was not explored in the current study. Instead, we chose to focus on SOCE.

The measured SOCE is dependent on the Ca^2+^ current carried by heteromeric CRAC channels formed by varying ratios of Orai1, 2, and 3 subunits (49, 50). This arrangement enables the CRAC channel activity and thus the SOCE magnitude to be tuned by adjusting the expression of Orai isoform subunits. For example, increasing Orai1 or 3 relative to Orai2 increases CRAC current and SOCE, while increasing Orai2 relative to Orai1 or 3 tends to be inhibitory and decrease SOCE (51, 52). Additional regulation is conferred by the stoichiometry of Orai and STIM proteins, such that increasing STIM without a complementary increase in the number of CRAC channels fails to increase SOCE (53, 54). Furthermore, upregulation of STIM2 has been shown to inhibit SOCE, suggesting that modifying the relative expression of STIM1 and 2 is yet another mechanism of regulation (55). Clearly, the relationship between SOCE and *Orai* and *STIM* isoform expression is complex. In our experiments, the effect of inflammatory phenotype on SOCE gene expression is qualitatively similar in both males and females (Fig. 2*A*). We see upregulation of the *Orai1*, and *2* genes during M1 and M2 stimulation and *STIM2* being upregulated by M1 stimulation. It is possible that these expression changes are sufficient to produce the observed changes in SOCE with M1 and M2 stimulation, however, without actual protein quantification and information on stoichiometry, this interpretation is highly speculative. Nevertheless, our data are consistent with the hypothesis that SOCE genes are exquisitely regulated by macrophage phenotype.

Sex differences in Orai and STIM expression were previously reported in the vasculature of a spontaneously hypertensive rat model, however, no direct measurements of SOCE were made (56). In addition, Orai and STIM expression levels were shown to be sensitive to sex hormone regulation in cancer cell lines (57, 58). We report that SOCE is lower in female macrophages compared to males, as demonstrated in Figure 1*E*. The persistence of these sex differences in BMDMs which have been differentiated and cultured without the addition of sex hormones, suggests that these characteristics are established early in the cell's developmental program. Furthermore, the fact that similar differences are observed in freshly isolated ATMs and monocytes indicates that these differences are not merely artifacts of cell culture (Figs. 2, *C* and *D* and 7, *A* and *D*). Not surprisingly, mRNA expression levels of Orai and STIM isoforms also differ between males and females (Fig. 2).

To explore the mechanistic consequences of SOCE sex differences, we generated a STIM1 knockout model (STIM1-KO). The complete loss of Ca^2+^ influx in store-depleted STIM1-KO cells demonstrates the requirement for STIM1 in activating SOCE in macrophages (Fig. 3, *B* and *C*). Measurement of the filing state of the endoplasmic reticulum (ER) Ca^2+^ stores also revealed a sex difference (Fig. 3*D*). As expected, males possess a higher stored Ca^2+^ content than females, consistent with males having larger SOCE. However, knocking out STIM1 affected Ca^2+^ store content in the male-only, decreasing store content to levels similar to those seen in the female. These data suggest that a STIM1-dependent mechanism normally operates to supplement stored Ca^2+^ in males and that this mechanism is absent in females. The molecular nature of such a mechanism remains to be defined.

The complement C5a receptor signals through G***α***_i_-coupled heterotrimers to evoke IP_3_R-dependent ER Ca^2+^ release and subsequent SOCE activation (36, 37, 59). C5a-evoked Ca^2+^ transients are larger in males compared to females (Fig. 3, *F* and *G*). Once again, the sex differences are eliminated by STIM1 knockout, although additional experiments are needed to determine if STIM1 dependence is mediated through decreased Ca^2+^ influx or reduced ER store content. Nevertheless, we can conclude that deleting STIM1 equalizes the C5a-dependent Ca^2+^ signals in males and females by eliminating SOCE and normalizing ER store content.

STIM1 knockout also equalizes chemotaxis in males and females (Fig. 4) by a mechanism that is dependent on STIM1-regulated C5a-evoked Ca^2+^ signaling. These findings appear at odds with previous studies of peritoneal macrophages that report C5a-dependent Ca^2+^ signaling to be dispensable for chemotaxis and independent of STIM1 (60, 61). Indeed, peritoneal macrophages SOCE was shown by Sogkas *et al.* to be dependent on both STIM1 and STIM2 (60), whereas STIM1 appears to be the dominant Ca^2+^ sensor in the current study. This disparity could be attributed to peritoneal macrophages being resident cells, and therefore likely to have adopted a completely different phenotype to that of BMDMs. In addition, our chemotaxis experiments were performed on fibronectin-coated coverslips which are known to enhance chemotaxis by promoting adhesion through Ca^2+^ signaling-dependent integrin-fibronectin interactions (62, 63, 64). Additionally, Sogkas *et al.* reported STIM1 knockout to have no effect on macrophage chemotaxis. However, their study used chimeric mice made by transplanting bone marrow from systemic knockouts into irradiated wild-type female recipients, and so, macrophages would have matured on a female background, regardless of donor sex.

Cells move in the direction of membrane extensions at the leading edge, where membrane dynamics are influenced by spatially and temporally regulated Ca^2+^ signals (14, 15, 65). In macrophages, the leading edge is maintained by Ca^2+^-dependent signaling that coordinates actin localization, however, the identity of the channels involved is not known (16, 66). We hypothesized a role for STIM1-mediated SOCE, however, deleting STIM1 had no effect on lamellipodia or ruffles in either male or female BMDMs, demonstrating that SOCE is not required to maintain leading edge membrane dynamics (Fig. 5, *A–D*). Interestingly, we did observe an increased frequency of lamellipodia and ruffles in females compared to males. The frequency of membrane protrusions is also an important determinant of phagocytic ability in macrophages (67); therefore, our observed sex difference in lamellipodia and ruffles might reflect the well-established increased phagocytic capacity of female macrophages (68, 69).

Chemotaxis is also regulated by trailing edge membrane dynamics. The formation of an elongated tail in chemotaxing cells creates physical drag which aids in directional persistence (70). Proper tail formation and retraction are coordinated by the assembly and disassembly of focal adhesions (FAs) (13). Increased actomyosin contractility driven by Rho-ROCK signaling at the rear of the cell promotes the disassembly of FAs, facilitating tail retraction (71). Impaired FA disassembly and trailing edge detachment are known to increase tail length and decrease directionality (72). We determined that in female cells, tails were longer in length, formed and retracted more slowly, and persisted longer. Deletion of STIM1 in male cells remodeled trailing edge dynamics to resemble that of the female cells (Fig. 5, *E* and *F*). Taken together, we show that STIM1 deletion in males eliminates sex differences in chemotaxis (Fig. 4) by modulating trailing edge dynamics (Fig. 5, *G–F*). Additional studies are needed to identify the downstream targets of STIM1. These are likely to be Ca^2+^-sensitive components or regulators of FAs. Indeed, in HEK293 cells, STIM1 accumulates at FAs where it promotes disassembly and tail retraction possibly influencing Rho-ROCK signaling; moreover, siRNA knockdown of STIM1 inhibits disassembly and increases tail length (28). The effects of STIM1 are most likely mediated through its role in gating Orai channels and regulating Ca^2+^ signaling. In the current study, the involvement of this pathway is evidenced by the ability of BTP2, an Orai channel inhibitor, to recapitulate the SOCE and chemotaxis phenotype (Fig. 6).

Males are more susceptible than females to a range of infectious diseases and generally have worse outcomes (73). The disparity results in part from reduced leukocyte mobilization and infiltration in females. In a study by Kay *et al.*, males exhibited increased neutrophil and monocyte recruitment to the peritoneal cavity in response to zymosan-induced inflammation. Moreover, they showed that infiltration of adoptively transferred male monocytes was greater than female monocytes, regardless of the recipient's sex (3). We also see the same-sex difference in WT cells using a similar experimental paradigm (Fig. 7). Knockout of STIM1, however, eliminated the sex difference by reducing the infiltration of male donor cells (Fig. 7*F*). These data add new mechanistic insight by demonstrating a key role for STIM1-dependent SOCE in mediating sex differences in monocyte recruitment.

In conclusion, we have identified STIM1-mediated SOCE and its resultant effect on the trailing edge as a novel mechanism that confers sex differences in monocyte recruitment and macrophage chemotaxis and defined its relationship to inflammatory phenotype. Our findings provide insight into inflammatory conditions characterized by sex differences in monocyte/macrophage infiltration. These include both acute conditions such as infection and trauma (3, 6), but also chronic diseases associated with obesity-induced adipose tissue inflammation (5, 74). Overall, our studies further highlight the importance of sex as a consideration in defining physiological mechanisms and future therapeutic interventions.

## Experimental procedures

### Chemicals and reagents

Recombinant mouse IL-4, recombinant mouse IFNγ, recombinant mouse complement C5a (R & D systems, Inc); CPA (Alfa Aesar); ethylene glycol-bis(β-aminoethyl ether)-N,N,N′,N′-tetraacetic acid (EGTA; J.T. Baker); fibronectin (Sigma-Aldrich); ACK buffer (Quality Biological Inc); Fetal Bovine Serum (FBS; Gemini Bio Products); recombinant mouse M-CSF (Tonbo Biosciences); Dulbecco's Modified Eagle Medium (DMEM), N-[4-[3,5-Bis(trifluoromethyl)-1H-pyrazol-1-yl]phenyl]-4-methyl-1,2,3-thiadiazole-5-carboxamide (BTP2; Bio-Techne Corporation), CytoTrace Green CMFDA (AAT Bioquest, Inc), Ham's F-12, antibiotic/antimycotic (Corning Inc - Life Sciences); fura2-AM (Biotium); probenecid, M-MLV Reverse Transcriptase (Thermo Fisher Scientific Corp); Trizol (Applied Biosystems), and Syber Green PCR Master Mix (Applied Biosystems), MojoSort Mouse Monocyte Isolation Kit, CD11b clone M1-70 and CD45 clone S18009F (Biolegend).

### Animals

Male and female C57BL/6J mice were purchased from The Jackson Laboratory and housed in the biological resource facility at Rosalind Franklin University. For the study duration, all mice were housed with a 12-h light/dark cycle in 12 × 6.25-inch cages with standard enrichment and *ad libitum* access to food and water. Euthanasia was by inhalation of a lethal dose of CO-_2_ followed by cervical dislocation, according to the National Institutes of Health Guide for the Care and Use of Laboratory Animals and approved by the Institutional Animal Care and Use Committee of Rosalind Franklin University of Medicine and Science. STIM1 hematopoietic KO mice were generated using B6.Cg-*Stim1*^*tm1Rao*^/J (STIM1^fl/fl^) (75) which were the kind gift of Anne George (University of Illinois Chicago) and B6.Cg-*Commd10*^*Tg(Vav1-icre)A2Kio*^/J JAX stock number 008610 purchased from The Jackson Laboratory. Mice were crossed to generate Vav-cre^+^ STIM1^fl/WT^ progeny which were then backcrossed to the parent generation to generate homozygous Vav-cre^+^ STIM1^fl/fl^ mice. The line was maintained by a Vav-cre^+^ STIM1^fl/fl^ × Vav-cre^−^ STIM1^fl/fl^ breeding scheme. Stim1fl/fl Vav1-Cre age-matched littermates were used as controls.

### Bone marrow-derived and adipose tissue macrophages (BMDMs and ATMs)

For BMDMs, femur and tibia bones were dissected from 5- to 10-week-old male and female mice, and the marrow was extracted by centrifugation for 30 s at 14,000 RPM, or by removing the epiphyses and flushing the marrow out with a 23G needle. The pellet was resuspended in ACK buffer for 2 min followed by the addition of Ca^2+^ and Mg^2+^ free phosphate-buffered saline (PBS) containing 1% FBS. The cells were then resuspended in BMDM complete culture media comprising 1:1 DMEM and Ham's F-12 supplemented with 10% FBS, 1% antibiotic/antimycotic, and 20 ng/ml recombinant mouse M-CSF and plated onto a 10 cm uncoated tissue culture dish and incubated overnight under tissue culture conditions. The following day, cells were split evenly into three wells of a tissue culture coated 6-well plate and cultured for a further 7 days before harvesting. For ATMs, the stromal vascular fraction was first isolated from gonadal fat pads, as described (76). The macrophage population within the SVF was then purified by immunomagnetic separation (EasySep Mouse CD11b Positive Selection Kit II; STEMCELL Technologies Inc) according to the manufacturer’s protocol.

### Cytoplasmic calcium ([Ca^2+^]_cyto_) measurement

BMDMs suspended in complete media were seeded at 4.0 × 10^5^ cells per well in a 24-well plate containing round 11 mm glass coverslips and allowed to adhere for at least 2 h. Cells were then loaded with 2 μM fura2-AM for 45 min at room temperature and the coverslip was mounted in a recording chamber and positioned on the stage of an IX71 inverted fluorescence microscope (Olympus Corp). A CCD-based imaging system running SimplePCI software (Hamamatsu Photonics) was used to record fura-2 fluorescence and the [Ca^2+^]_cyto_ reported as the ratio of background-corrected fluorescence intensity excited at 340 nm and 380 nm and collected at 510 nm. Experiments that measured SOCE were performed at room temperature and cells were continuously perfused with Hank’s Balanced Salt Solution (HBSS) containing (mM): NaCl (137.9), KCl (5.33), KH_2_PO_4_ (0.44), Na_2_HPO_4_ (0.34), Glucose (5.56), NaHCO_3_ (4.17), CaCl_2_ (1.8), MgCl_2_ (0.49), MgSO_4_ (0.41), HEPES (10), pH 7.4 with NaOH. To prepare Ca^2+^-free HBSS, CaCl_2_ was substituted with MgCl_2_ and 1 mM EGTA was added. For experiments that measured [Ca^2+^]_cyto_ in response to the addition of Complement C5a, cells were maintained at 37 °C in the continued presence of 1 mM probenecid, a blocker of organic anion transporters that reduces loss of fura-2 (77).

### Chemotaxis

Chemotaxis was assayed using the μ-Slide Chemotaxis system (Ibidi GmbH), according to the manufacturer’s instructions, and as described (31). Each μ-Slide has two solution reservoirs on either side of a central chamber. Filling one of the chambers with chemoattractant generates a concentration across the central chamber. Cells cultured in the central chamber are then visualized moving toward the chemoattractant-containing reservoir. Prior to loading cells, the μ-slides were coated with fibronectin (40 μg/ml) in PBS, for 2 h at 37 °C and washed three times with PBS. Cells were then loaded into the central chamber and allowed to adhere for 2 h in a tissue culture incubator. To establish the chemotactic gradient, one of the reservoirs was filled with culture media (60 μl) and the other with culture media containing 20 nM recombinant mouse complement C5a. The loaded slide was then placed in a custom-made incubator and maintained at 37 °C in a humidified 5% CO_2_ atmosphere on the stage of a Nikon Eclipse TE2000-U (Nikon Instruments Inc) and cells visualized using a ×10/0.45 DIC objective. Images were acquired every 2 min using MetaMorph Microscopy Automation and Image Analysis Software (Molecular Devices, LLC). Images captured between 2 and 9 h were analyzed using the FastTrack AI software (MetaVi Labs Inc) to define individual cell migration tracks, and the chemotaxis and migration plugin (Ibidi) for ImageJ (31, 78) to generate migration plots and calculate Forward Migrating Index (FMI_Δx_).

### Membrane dynamics

BMDMs (5.0 × 10^5^ cells/ml) were plated on fibronectin (10 μg/ml) coated coverslips and mounted in a chamber on the microscope stage (Nikon Eclipse TE2000-U). Cells were visualized using a ×40/0.95 DIC objective and the preparation was continuously perfused with HBSS at 37 °C. A glass micropipette containing C5a (10 nM) coupled to a homemade picospritzer was positioned 30 to 40 μm from the cell and a localized concentration gradient was established by delivering 20 ms pulses of C5a every second. Images were captured every 2 s for 10 min using MetaMorph Microscopy Automation and Image Analysis Software. Recordings were further analyzed in ImageJ. First, the line tool was used to mark a single pixel-wide line from the cell nucleus to the pipette tip. Next, the pixel-wide images were extracted from the same position at each time point and aligned as a space-time plot using the KymographBuilder plugin (79). The kymograph plots were used to quantify lamellipodia extensions and ruffles as previously described in detail (39).

### Real-time quantitative PCR and Western blot

Total RNA was extracted from BMDMs using Trizol according to the manufacturer’s protocol and cDNA was synthesized from 1 μg of total RNA using M-MLV Reverse Transcriptase. Real-time quantitative RT-PCR was performed on a ViiA7 thermo cycler using Syber Green PCR Master Mix and validated primers. Fluorescent signals generated during PCR amplifications were normalized to an internal reference (Hprt), the threshold cycle (Ct) was set within the exponential phase, and the relative quantitative evaluation of target gene levels was performed using the 2^−ΔΔCt^ method. For Western blot, whole cell lysates were prepared in RIPA buffer and a protease inhibitor cocktail, resolved by SDS-PAGE in a 10% gel, and transferred to a nitrocellulose membrane. Membranes were blocked with 5% non-fat milk in TBST (10 mM Tris, pH 8.0, 150 mM NaCl, 0.5% Tween 20) for 60 min, incubated with primary antibodies for 12 h at 4 °C, then washed with TBST three times and incubated with HRP-conjugated secondary antibodies for 60 min at room temperature. The membrane was washed three times and developed using the ECL system. Primary antibodies used included Anti-GOK/Stim1 (1:250), BD Transduction Laboratories; anti-Actin (1:1000), Sigma Life Science (A2066-2Ml).

### *In vivo* monocyte trafficking

Bone marrow cells were harvested as described above and monocytes were isolated using MojoSort Mouse Monocyte Isolation Kit according to the manufacturer’s instructions. Monocytes were resuspended in FACS buffer (PBS supplemented with 2% FBS, and 1 mM EDTA) and labeled with CytoTrace Green CMFDA (0.2 μM) for 15 min at 37 °C. Cells were washed once and resuspended in sterile PBS. Recipient animals were first i.p.-injected with 0.5 ml zymosan (1 mg/ml) followed 30 min later by an i.v.-injection of 2 × 10^6^ labeled monocytes. Peritoneal cells were harvested 18 h later and stained with antibodies specific for CD11b and CD45 and analyzed by flow cytometry.

### Data collection and statistical analysis

For μ-Slide Chemotaxis experiments we tracked 2 to 200 cells per well to generate one set of the center of mass coordinates, Forward Migration Index (FMI_Δx_), velocity, and directionality. The number of independent replicates and animals used are indicated in the figure legends. Calcium imaging measurements were made on individual cells. The number of cells, coverslips, and animals are indicated on the figure legend. We summarized the data as box (median and 25th to 75th percentiles) and whisker (10th and 90th percentile) plots. A Student’s *t* test was used for pairwise comparisons and a one-way ANOVA with Bonferroni *post hoc* analysis was used for multiple comparisons. The effect of, and interaction between, sex and inflammatory phenotype (M0, M1, M2), or genotype (WT, STIM-KO), was assessed by two-way ANOVA. Analyses were performed using GraphPad Prism version 9, and *p* < 0.05 was considered significant.

## Data availability

All data are contained within the manuscript. Analyzed data files are available from the corresponding author on request.

## Supporting information

This article contains supporting information.

## Conflict of interest

The authors declare that they have no known competing financial interests or personal relationships that could have appeared to influence the work reported in this paper.

## Supporting information

Supplementary Video 3

Supplementary Videos 1–3 Caption

## References

[1] Gal-Oz S.T., Maier B., Yoshida H., Seddu K., Elbaz N., Czysz C. (2019). ImmGen report: sexual dimorphism in the immune system transcriptome. Nat. Commun..

[2] Klein S.L., Flanagan K.L. (2016). Sex differences in immune responses. Nat. Rev. Immunol..

[3] Kay E., Gomez-Garcia L., Woodfin A., Scotland R.S., Whiteford J.R. (2015). Sexual dimorphisms in leukocyte trafficking in a mouse peritonitis model. J. Leukoc. Biol..

[4] Chen K.-H.E., Lainez N.M., Coss D. (2020). Sex differences in macrophage responses to obesity-mediated changes determine migratory and inflammatory traits. J. Immunol..

[5] Varghese M., Clemente J., Lerner A., Abrishami S., Islam M., Subbaiah P. (2022). Monocyte trafficking and polarization contribute to sex differences in meta-inflammation. Front. Endocrinol..

[6] Doran S.J., Ritzel R.M., Glaser E.P., Henry R.J., Faden A.I., Loane D.J. (2019). Sex differences in acute neuroinflammation after experimental traumatic brain injury are mediated by infiltrating myeloid cells. J. Neurotrauma.

[7] Jones G.E. (2000). Cellular signaling in macrophage migration and chemotaxis. J. Leukoc. Biol..

[8] Li K., Xu W., Guo Q., Jiang Z., Wang P., Yue Y. (2009). Differential macrophage polarization in male and female BALB/c mice infected with coxsackievirus B3 defines susceptibility to viral myocarditis. Circ. Res..

[9] Melgert B.N., Oriss T.B., Qi Z., Dixon-McCarthy B., Geerlings M., Hylkema M.N. (2010). Macrophages: regulators of sex differences in asthma?. Am. J. Respir. Cell Mol. Biol..

[10] Vogel D.Y.S., Heijnen P.D.A.M., Breur M., de Vries H.E., Tool A.T.J., Amor S. (2014). Macrophages migrate in an activation-dependent manner to chemokines involved in neuroinflammation. J. Neuroinflammation.

[11] Xuan W., Qu Q., Zheng B., Xiong S., Fan G.-H. (2015). The chemotaxis of M1 and M2 macrophages is regulated by different chemokines. J. Leukoc. Biol..

[12] Hind L.E., Lurier E.B., Dembo M., Spiller K.L., Hammer D.A. (2016). Effect of M1-M2 polarization on the motility and traction stresses of primary human macrophages. Cell. Mol. Bioeng..

[13] Ridley A.J. (2011). Life at the leading edge. Cell.

[14] Tsai F.-C., Meyer T. (2012). Ca2+ pulses control local cycles of lamellipodia retraction and adhesion along the front of migrating cells. Curr. Biol..

[15] Wei C., Wang X., Chen M., Ouyang K., Song L.-S., Cheng H. (2009). Calcium flickers steer cell migration. Nature.

[16] Evans J.H., Falke J.J. (2007). Ca2+ influx is an essential component of the positive-feedback loop that maintains leading-edge structure and activity in macrophages. Proc. Natl. Acad. Sci. U. S. A..

[17] Clark A.J., Petty H.R. (2008). Observation of calcium microdomains at the uropod of living morphologically polarized human neutrophils using flash lamp-based fluorescence microscopy. Cytometry A.

[18] Eddy R.J., Pierini L.M., Matsumura F., Maxfield F.R. (2000). Ca2+-dependent myosin II activation is required for uropod retraction during neutrophil migration. J. Cell Sci..

[19] Prakriya M., Lewis R.S. (2015). Store-operated calcium channels. Physiol. Rev..

[20] Feske S., Wulff H., Skolnik E.Y. (2015). Ion channels in innate and adaptive immunity. Annu. Rev. Immunol..

[21] Tsai F.-C., Seki A., Yang H.W., Hayer A., Carrasco S., Malmersjö S. (2014). A polarized Ca2+, diacylglycerol and STIM1 signalling system regulates directed cell migration. Nat. Cell Biol..

[22] Steinckwich N., Myers P., Janardhan K.S., Flagler N.D., King D., Petranka J.G. (2015). Role of the store-operated calcium entry protein, STIM1, in neutrophil chemotaxis and infiltration into a murine model of psoriasis-inflamed skin. FASEB J..

[23] Nunes-Hasler P., Maschalidi S., Lippens C., Castelbou C., Bouvet S., Guido D. (2017). STIM1 promotes migration, phagosomal maturation and antigen cross-presentation in dendritic cells. Nat. Commun..

[24] Chen Y.-W., Chen Y.-F., Chiu W.-T., Chen H.-C., Shen M.-R. (2017). STIM1-dependent Ca(2+) signaling regulates podosome formation to facilitate cancer cell invasion. Sci. Rep..

[25] Bisaillon J.M., Motiani R.K., Gonzalez-Cobos J.C., Potier M., Halligan K.E., Alzawahra W.F. (2010). Essential role for STIM1/Orai1-mediated calcium influx in PDGF-induced smooth muscle migration. Am. J. Physiol. Cell Physiol..

[26] Vashisht A., Trebak M., Motiani R.K. (2015). STIM and Orai proteins as novel targets for cancer therapy. A review in the theme: cell and molecular processes in cancer metastasis. Am. J. Physiol. Cell Physiol..

[27] Lopez-Guerrero A.M., Espinosa-Bermejo N., Sanchez-Lopez I., Macartney T., Pascual-Caro C., Orantos-Aguilera Y. (2020). RAC1-Dependent ORAI1 translocation to the leading edge supports lamellipodia formation and directional persistence. Sci. Rep..

[28] Schäfer C., Rymarczyk G., Ding L., Kirber M.T., Bolotina V.M. (2012). Role of molecular determinants of store-operated Ca(2+) entry (Orai1, phospholipase A2 group 6, and STIM1) in focal adhesion formation and cell migration. J. Biol. Chem..

[29] Jablonski K.A., Amici S.A., Webb L.M., Ruiz-Rosado J.D., Popovich P.G., Partida-Sanchez S. (2015). Novel markers to delineate murine M1 and M2 macrophages. PLoS One.

[30] Ying W., Cheruku P.S., Bazer F.W., Safe S.H., Zhou B. (2013). Investigation of macrophage polarization using bone marrow derived macrophages. J. Vis. Exp..

[31] Zengel P., Nguyen-Hoang A., Schildhammer C., Zantl R., Kahl V., Horn E. (2011). μ-Slide Chemotaxis: a new chamber for long-term chemotaxis studies. BMC Cell Biol..

[32] Petrie Aronin C.E., Zhao Y.M., Yoon J.S., Morgan N.Y., Prüstel T., Germain R.N. (2017). Migrating myeloid cells sense temporal dynamics of chemoattractant concentrations. Immunity.

[33] Ward P.A. (2004). The dark side of C5a in sepsis. Nat. Rev. Immunol..

[34] Sogkas G., Vögtle T., Rau E., Gewecke B., Stegner D., Schmidt R.E. (2015). Orai1 controls C5a-induced neutrophil recruitment in inflammation. Eur. J. Immunol..

[35] Monk P.N., Partridge L.J. (1993). Characterization of a complement-fragment-C5a-stimulated calcium-influx mechanism in U937 monocytic cells. Biochem. J..

[36] Jiang H., Kuang Y., Wu Y., Smrcka A., Simon M.I., Wu D. (1996). Pertussis toxin-sensitive activation of phospholipase C by the C5a and fMet-Leu-Phe receptors. J. Biol. Chem..

[37] Möller T., Nolte C., Burger R., Verkhratsky A., Kettenmann H. (1997). Mechanisms of C5a and C3a complement fragment-induced [Ca2+]i signaling in mouse microglia. J. Neurosci..

[38] Schwab A., Fabian A., Hanley P.J., Stock C. (2012). Role of ion channels and transporters in cell migration. Physiol. Rev..

[39] Borm B., Requardt R.P., Herzog V., Kirfel G. (2005). Membrane ruffles in cell migration: indicators of inefficient lamellipodia adhesion and compartments of actin filament reorganization. Exp. Cell Res..

[40] He L.-P., Hewavitharana T., Soboloff J., Spassova M.A., Gill D.L. (2005). A functional link between store-operated and TRPC channels revealed by the 3,5-bis(trifluoromethyl)pyrazole derivative, BTP2. J. Biol. Chem..

[41] Cash J.L., White G.E., Greaves D.R. (2009). Chapter 17. Zymosan-induced peritonitis as a simple experimental system for the study of inflammation. Methods Enzymol..

[42] Yanguas-Casás N., Crespo-Castrillo A., de Ceballos M.L., Chowen J.A., Azcoitia I., Arevalo M.A. (2018). Sex differences in the phagocytic and migratory activity of microglia and their impairment by palmitic acid. Glia.

[43] Yano H., Uchida M., Saito T., Aoki T., Kremenik M.J., Oyanagi E. (2018). Reduction of real-time imaging of M1 macrophage chemotaxis toward damaged muscle cells is PI3K-dependent. Antioxidants (Basel).

[44] Uchida M., Oyanagi E., Miyachi M., Yamauchi A., Yano H. (2013). Relationship between macrophage differentiation and the chemotactic activity toward damaged myoblast cells. J. Immunol. Methods.

[45] Cui K., Ardell C.L., Podolnikova N.P., Yakubenko V.P. (2018). Distinct migratory properties of M1, M2, and resident macrophages are regulated by αDβ2 and αMβ2 integrin-mediated adhesion. Front. Immunol..

[46] Gouwy M., Struyf S., Leutenez L., Pörtner N., Sozzani S., Van Damme J. (2014). Chemokines and other GPCR ligands synergize in receptor-mediated migration of monocyte-derived immature and mature dendritic cells. Immunobiology.

[47] Liu Z., Jiang Y., Li Y., Wang J., Fan L., Scott M.J. (2013). TLR4 Signaling augments monocyte chemotaxis by regulating G protein-coupled receptor kinase 2 translocation. J. Immunol..

[48] Aomatsu K., Kato T., Fujita H., Hato F., Oshitani N., Kamata N. (2008). Toll-like receptor agonists stimulate human neutrophil migration via activation of mitogen-activated protein kinases. Immunology.

[49] Emrich S.M., Yoast R.E., Xin P., Arige V., Wagner L.E., Hempel N. (2021). Omnitemporal choreographies of all five STIM/Orai and IP3Rs underlie the complexity of mammalian Ca2+ signaling. Cell Rep..

[50] Yoast R.E., Emrich S.M., Zhang X., Xin P., Johnson M.T., Fike A.J. (2020). The native ORAI channel trio underlies the diversity of Ca2+ signaling events. Nat. Commun..

[51] Vaeth M., Yang J., Yamashita M., Zee I., Eckstein M., Knosp C. (2017). ORAI2 modulates store-operated calcium entry and T cell-mediated immunity. Nat. Commun..

[52] Tsvilovskyy V., Solís-López A., Schumacher D., Medert R., Roers A., Kriebs U. (2018). Deletion of Orai2 augments endogenous CRAC currents and degranulation in mast cells leading to enhanced anaphylaxis. Cell Calcium.

[53] McNally B.A., Somasundaram A., Yamashita M., Prakriya M. (2012). Gated regulation of CRAC channel ion selectivity by STIM1. Nature.

[54] Scrimgeour N., Litjens T., Ma L., Barritt G.J., Rychkov G.Y. (2009). Properties of Orai1 mediated store-operated current depend on the expression levels of STIM1 and Orai1 proteins. J. Physiol..

[55] Miederer A.-M., Alansary D., Schwär G., Lee P.-H., Jung M., Helms V. (2015). A STIM2 splice variant negatively regulates store-operated calcium entry. Nat. Commun..

[56] Giachini F.R.C., Lima V.V., Filgueira F.P., Dorrance A.M., Carvalho M.H.C., Fortes Z.B. (2012). STIM1/Orai1 contributes to sex differences in vascular responses to calcium in spontaneously hypertensive rats. Clin. Sci..

[57] Liu G., Honisch S., Liu G., Schmidt S., Alkahtani S., AlKahtane A.A. (2015). Up-regulation of Orai1 expression and store operated Ca(2+) entry following activation of membrane androgen receptors in MCF-7 breast tumor cells. BMC Cancer.

[58] Motiani R.K., Abdullaev I.F., Trebak M. (2010). A novel native store-operated calcium channel encoded by Orai3: selective requirement of Orai3 versus Orai1 in estrogen receptor-positive versus estrogen receptor-negative breast cancer cells. J. Biol. Chem..

[59] Särndahl E., Bokoch G.M., Boulay F., Stendahl O., Andersson T. (1996). Direct or C5a-induced activation of heterotrimeric Gi2 proteins in human neutrophils is associated with interaction between formyl peptide receptors and the cytoskeleton. J. Biol. Chem..

[60] Sogkas G., Stegner D., Syed S.N., Vögtle T., Rau E., Gewecke B. (2015). Cooperative and alternate functions for STIM1 and STIM2 in macrophage activation and in the context of inflammation. Immun. Inflamm. Dis..

[61] van den Bos E., Ambrosy B., Horsthemke M., Walbaum S., Bachg A.C., Wettschureck N. (2020). Knockout mouse models reveal the contributions of G protein subunits to complement C5a receptor-mediated chemotaxis. J. Biol. Chem..

[62] Hsieh J.Y., Keating M.T., Smith T.D., Meli V.S., Botvinick E.L., Liu W.F. (2019). Matrix crosslinking enhances macrophage adhesion, migration, and inflammatory activation. APL Bioeng..

[63] Tvorogov D., Wang X.-J., Zent R., Carpenter G. (2005). Integrin-dependent PLC-gamma1 phosphorylation mediates fibronectin-dependent adhesion. J. Cell Sci..

[64] Park J.H., Ryu J.M., Yun S.P., Kim M.O., Han H.J. (2012). Fibronectin stimulates migration through lipid raft dependent NHE-1 activation in mouse embryonic stem cells: involvement of RhoA, Ca(2+)/CaM, and ERK. Biochim. Biophys. Acta.

[65] Arrieumerlou C., Meyer T. (2005). A local coupling model and compass parameter for eukaryotic chemotaxis. Dev. Cell..

[66] Ziemba B.P., Falke J.J. (2018). A PKC-MARCKS-PI3K regulatory module links Ca2+ and PIP3 signals at the leading edge of polarized macrophages. PLoS One.

[67] Paterson N., Lämmermann T. (2022). Macrophage network dynamics depend on haptokinesis for optimal local surveillance. Elife.

[68] Gay L., Melenotte C., Lopez A., Desnues B., Raoult D., Leone M. (2021). Impact of sex hormones on macrophage responses to coxiella burnetii. Front. Immunol..

[69] Scotland R.S., Stables M.J., Madalli S., Watson P., Gilroy D.W. (2011). Sex differences in resident immune cell phenotype underlie more efficient acute inflammatory responses in female mice. Blood.

[70] Theisen U., Straube E., Straube A. (2012). Directional persistence of migrating cells requires Kif1C-mediated stabilization of trailing adhesions. Dev. Cell..

[71] Ridley A.J., Schwartz M.A., Burridge K., Firtel R.A., Ginsberg M.H., Borisy G. (2003). Cell migration: integrating signals from front to back. Science.

[72] Liu J., Liu Z., Chen K., Chen W., Fang X., Li M. (2021). Kindlin-2 promotes rear focal adhesion disassembly and directional persistence during cell migration. J. Cell Sci..

[73] Marriott I., Huet-Hudson Y.M. (2006). Sexual dimorphism in innate immune responses to infectious organisms. Immunol. Res..

[74] Singer K., Maley N., Mergian T., DelProposto J., Cho K.W., Zamarron B.F. (2015). Differences in hematopoietic stem cells contribute to sexually dimorphic inflammatory responses to high fat diet-induced obesity. J. Biol. Chem..

[75] Oh-Hora M., Yamashita M., Hogan P.G., Sharma S., Lamperti E., Chung W. (2008). Dual functions for the endoplasmic reticulum calcium sensors STIM1 and STIM2 in T cell activation and tolerance. Nat. Immunol..

[76] Orr J.S., Kennedy A.J., Hasty A.H. (2013). Isolation of adipose tissue immune cells. J. Vis. Exp..

[77] Di Virgilio F., Steinberg T.H., Swanson J.A., Silverstein S.C. (1988). Fura-2 secretion and sequestration in macrophages. A blocker of organic anion transport reveals that these processes occur via a membrane transport system for organic anions. J. Immunol..

[78] Schindelin J., Arganda-Carreras I., Frise E., Kaynig V., Longair M., Pietzsch T. (2012). Fiji: an open-source platform for biological-image analysis. Nat. Methods.

[79] Mary H., Rueden C., Ferreira T. (2016). KymographBuilder: release 1.2.4. Zenodo.

