# Supplementary material for: STIM1-dependent store-operated calcium entry mediates sex differences in macrophage chemotaxis and monocyte recruitment
Source: J Biol Chem. 2024 May 28;300(7):107422. doi: 10.1016/j.jbc.2024.107422 (PMC11231831; doi:10.1016/j.jbc.2024.107422)
Supplement: Supplementary Videos 1–3 Caption [file mmc4.pdf]

STIM1-Dependent Store-Operated Calcium Entry Mediates Sex Differences in Macrophage Chemotaxis and Monocyte Recruitment.

Adriana M. Fresquez, James O. Hogan, Patricia Rivera1, Kristen M. Patterson, Joseph M. Reynolds, Kanakadurga Singer and Carl White.

Supporting Video 1. Male bone marrow derived macrophages (BMDMs) chemotaxis. Left panel shows cells in a μ-Slide chemotaxis assay chamber undergoing chemotactic motility in concentration gradient C5a. Right panel shows the tracking plots detected by FastTrack AI software.

Supporting Video 2. Female bone marrow derived macrophages (BMDMs) chemotaxis. Left panel shows cells in a μ-Slide chemotaxis assay chamber undergoing chemotactic motility in concentration gradient C5a. Right panel shows the tracking plots detected by FastTrack AI software.

Supporting Video 3. Male bone marrow derived macrophages (BMDMs) chemotaxis. Bone marrow derived macrophages (BMDMs) responding to C5a delivered from a micropipette.
